# Indentation Size Effect in Pressure-Sensitive Polymer Based on A Criterion for Description of Yield Differential Effects and Shear Transformation-Mediated Plasticity

**DOI:** 10.3390/polym11030412

**Published:** 2019-03-04

**Authors:** Hui Lin, Tao Jin, Lin Lv, Qinglin Ai

**Affiliations:** 1 Key Laboratory of E&M, Ministry of Education & Zhejiang Province, Zhejiang University of Technology, Hangzhou 310014, China; linhuizipc@163.com; 2 Zhejiang Industry Polytechnic College, Shaoxing 312000, China; lvlinzipc@163.com; 3 Institute of Applied Mechanics, Taiyuan University of Technology, Taiyuan 030024, China; tyutjintao@163.com; 4 State Key Laboratory for Strength and Vibration of Mechanical Structures, Xi’an Jiaotong University, Xi’an 710049, China

**Keywords:** Pressure-sensitive polymer, indentation size effects, SD effects, yield criterion, shear transformation-mediated plasticity

## Abstract

Indentation size effects in poly(methyl methacrylate) (PMMA) were studied through nanoindentation. Two factors of indentation size effects in PMMA, namely yield criterion and shear transformation-mediated plasticity, were analysed in detail. The yield criterion that considers strength differential (SD) effects and pressure sensitivity was constructed by performing the combined shear-compression experiments. The relationship between hardness and normal stress can then be obtained based on Tabot’s relation. Shear transformation-mediated plasticity was also applied to model the measured hardness as a function of the indentation depth at different strain rates. Results show that the yield criterion contains the terms of SD effects and pressure sensitivity gives the best description of the yielding of PMMA. Additionally, the volume of single shear transformation zone calculated through the presented criterion agrees well with simulation and exhibits increases with increasing strain rate. Indentation size effects in PMMA under different strain rates were discussed and an appropriate indentation depth range was suggested for calculating the hardness and modulus.

## 1. Introduction

Poly(methyl methacrylate) (PMMA) is a widely used material in the field of aircraft and automotive industries due to its excellent properties such as transparency, low density, and high impact resistance [1,2,3,4]. Therefore, the mechanical behaviour of PMMA is an important property that should be clarified prior to its usage. Many studies have been conducted to investigate the macroscopic [5,6] and mesoscopic [7,8] mechanical properties of PMMA such as compression, tension, and nanoindentation. The yield strength and modulus of PMMA were found to be sensitive to strain rate and both properties exhibit increases with strain rate. From the compressive and tensile tests of PMMA, the strength of compression was found different from that of tension [9]. This phenomenon is the so-called strength differential (SD) effects, which results from the different deformation mechanisms (flow or fracture of molecular chain, crazing or formation of micro cavities), indicating that the yielding of PMMA is sensitive to the sign of normal stress. Furthermore, glass polymers (GPs) always exhibit pressure sensitivity of their yield and flow as metallic glasses (MGs) [10]. For MGs, if shear stress is applied, then collective rearrangement of clusters of small numbers of atoms, namely, shear transformation zone (STz). Then, some dilatation (irreversible volume increase) occurs at the microscopic level, which leads to the pressure sensitivity of yield. GPs are macromolecular materials with chain entanglement, they do not possess any long-range order and their plastic deformation is mediated by shear bands as MGs [11]. Therefore, the yield of GPs is also affected by pressure. Owing to the absence of long-range structural order in glassy solids, their flow and plastic response mechanism in the microstructure level are different from that of crystal plasticity, wherein dislocations are the main carriers of plasticity. These glide processes are only shear driven: neither significant dilatation nor any pressure sensitivity are at risk. Hence, the dislocations-mediated plasticity is inadequate for describing the yield and flow of GPs. As previously mentioned, STz mediates plasticity (the cooperative localised rearrangement of atomic or molecular clusters in small distinct regions). STz, which is a widely accepted mechanism for the plastic deformation in GPs, is initially applied to explain the highly localized heterogeneous deformation by formation of MG shear bands. Oleinik [12,13] has experimentally observed the formation of STz in GPs. Materials constantly used as a structural component in complex stress conditions, generally demands an overall understanding of the mechanical behaviour of material under complex stress states. Hence, investigating the mechanical properties of materials under combined loadings has drawn research interests for decades [14,15,16,17,18] and several test methods have been developed. Nie et al. [19] proposed to study the mechanical behaviour of material under combined shear–compression loading by conducting the uniaxial compression on a tilted specimen to obtain the additional shear stress. Analogously, Hou et al. [20,21] designed a combined shear–compression loading device comprising a short cylindrical bar system with one bevelled end (to acquire the additional shear stress) and used it to investigate the failure behaviour of aluminium honeycomb.

A constitutive equation is a powerful tool to describe or predict the mechanical behaviour of material under different stress states. However, some recent experiments on non-uniform plastic deformation have shown a size effect at the micro/nano scale [22]. A torsional experiment of copper wire with micron–diameter showed that the shear strength increases with reducing diameter [23], and the bending experiment of thin beam displayed that thinner beams have higher strength [24]. These experiments showed the evident size dependence of mechanical behaviour of materials. Therefore, the classical continuum plasticity cannot explain this situation because the constitutive equation of classical mechanics does not include constituent internal length as a deformation parameter [22]. Small–scale mechanical behaviour of materials is at the cutting edge of research in materials science and applied mechanics [25]. Nanoindentation testing is a widely used method for testing the mechanical properties of materials at small scales due to the improvements of instruments and the requirement of an understanding of how materials perform at small scales [26]. Moreover, the test method is usually non–destructive which means that it is promising for mechanical field testing. Many investigations have reported that the indentation hardness of materials exhibits a strong size dependence [27,28,29]. Nix and Gao [30] proposed a law for strain gradient plasticity to explain the indentation size effects in crystalline materials based on the approach of geometrically necessary dislocations and statistically stored dislocations. However, the deformation mechanism of PGs is different from that of crystalline materials as previously mentioned. Therefore, the size-dependent behaviours of PGs cannot be explained by dislocation-mediated plasticity. Some efforts have been made to attempt to explain the indentation size effects in GPs. Lam et al. [31] developed a strain gradient plasticity law on the basis of molecular theory of yield for GPs to model the depth dependence of hardness for thermosetting epoxy resin. By assuming the approach of statistically random kink pairs and geometrically necessary kink pairs, kink-pair yield theory, which is similar to the Nix and Gao model. Given that the main carrier of plasticity deformation in GPs is STz, the shear transformation-mediated plasticity is the widely accepted approach to model the indentation size effects in GPs as developed by Voyiadjis et al. [32]. 

Based on the two previously described aspects, two issues should be addressed before modelling the size effect behaviour of PMMA. On the one hand, a yield criterion, which reflects the multiaxial mechanical responses of PMMA, should be constructed. The criterion should have definite physical meaning to reflect the plastic deformation mechanism of amorphous materials as previously mentioned. In addition, the SD effects and pressure sensitivity should be independently introduced into the criterion. Thus, the effects of SD and pressure must be decoupled to avoid confusion unlike that in the previous investigation [15]. The combined loading tests should be conducted to identify the parameters in the criterion, and the method of tilted specimens without complex loading device [20,21] was selected for convenience. On the other hand, the volume of single STz should be calculated by applying the yield criterion and Tabor’s relation according to the shear transformation-mediated plasticity. The calculated volume of single STz clearly varies with the yield criterion, and constructing a reasonable criterion is crucial for the reliability of results. The indentation size effects of PMMA can then be modelled with comprehensive considerations of practical situation [32]. Consequently, the major aim of the present study can be summarised as modelling the indentation size effects in PMMA which can be divided into two main contents. Firstly, the yield criterion of PMMA, which contains the SD effects and pressure sensitivity, is constructed through combined loading tests. Secondly, the hardness and normal stress are correlated based on the criterion, and the volume of STz is then calculated to model the indentation size effects based on shear transformation-mediated plasticity. The experiment procedure of combined loading and nanoindentation is presented firstly in Section 2. The model of yield criterion and shear transformation-mediated plasticity of PMMA accompanied by some theoretical considerations is then introduced in Section 3. Section 4 displays the experimental results (combined loading and nanoindentation) and the derivation of model parameters (yield criterion and STz) from the experimental data and discusses of the indentation size effects in PMMA. Finally, conclusions are given in Section 5. 

## 2. Experiment Procedure

### 2.1. Materials and Specimens

Commercial grade of the material was purchased from Degussa AG Plexiglas^®^ PMMA (Shanghai, China). The PMMA sheet is produced through a traditional cell cast method, thus, no molecular chain orientation exists in the as-cast sheet.

**Figure 1:**
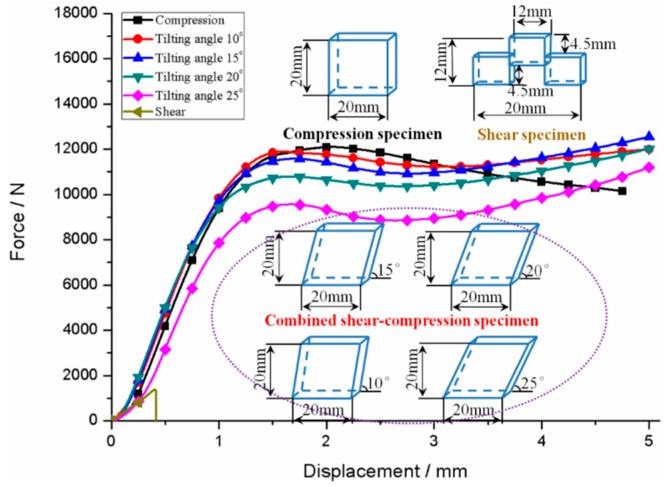
Force-displacement curves and schematic of poly(methyl methacrylate) (PMMA) specimens used in quasi-static loading.

The 20 × 20 mm square samples with thickness of 5 mm were used in the nanoindentation tests. The cube specimen, double shear specimen, and combined shear–compression specimens, that is, cube specimens with different tilting angles θ (10°, 15°, 20° and 25°) were applied in the quasi–static loading to obtain the yield loci of PMMA under different stress states as shown in Figure 1 This figure reveals the specified dimensions of PMMA specimens. The complex stress states (combined shear–compression) can be obtained due to the geometric effects which can be attributed to the existence of the tilting angle. Notably, the influence of the tilting angle of the PMMA specimens on the overall compressive response was investigated with a constant thickness of 5 mm.

### 2.2. Nanoindentation Tests

A nanoindenter test system (G200, Agilent Technologies, CA, USA) equipped with triangular pyramid Berkovich diamond indenter was employed to conduct the nanoindentation tests. The continuous stiffness measurement (CSM) technique was used in the nanoindentation tests. The continuous hardness and elastic modulus throughout the indentation process can then be obtained and the indentation tests were performed at different $\frac{\dot{P}}{P}$ of 0.05, 0.1, 0.2 and 0.3 s^−1^. Based on the CSM technique, an additional harmonic movement with a driving frequency of 45 Hz and an amplitude of 2 nm was applied on the indenter. The maximum indentation depth was 2000 nm and the indenter remained at the corresponding peak load for 10 s as long as the maximum indentation depth was achieved to release creep deformation. The load then dropped to 0.1P_max_ for thermal drift correction during the unloading stage. Furthermore, each test was performed at room temperature (25 °C) and repeated three to four times to exclude uncertain experimental results. For CSM technique, the contact stiffness *S* is expressed as [8,33,34]:

(1) $$S={\left[\frac{1}{\frac{F_{amp}}{h_{amp}}\cos\phi -\left(K_{s}-m\omega^{2}\right)}-\frac{1}{K_{f}}\right]}^{-1}$$

where *F*_amp_ and *h*_amp_ represent the amplitude of harmonic excitation force and the response displacement amplitude (~2 nm), respectively, ϕ is the phase shift that harmonic displacement lags behind the harmonic excitation force, ω=2πf is the angular frequency (*f* = 45 Hz), *K*_s_ is the spring constant in the vertical direction, *K*_f_ is frame stiffness, *m* is the mass of compression bar connected to the indenter. A perfect Berkovich diamond indenter was used in this study, and the projected contact area $A_{c}$ and contact depth $h_{c}$ can be respectively expressed as:

(2-1) $$A_{c}=24.56h_{c}^{2}$$

(2-2) $$h_{c}=h-\varepsilon \frac{P}{S}$$

where ε=0.75 is a constant for the Berkovich indenter. Then the hardness can be expressed as:

(3) $$H=\frac{P}{A_{c}}$$

where *P* represents the contact load. The elastic modulus of the indented material is obtained from the following equation [17]:

(4) $$\frac{1}{E_{r}}=\frac{1-\nu^{2}}{E}+\frac{1-\nu_{i}^{2}}{E_{i}}$$

where *E_i_*, $\nu_{i}$ are the modulus and Poisson’s ratio of the indenter tip (diamond), *E*, *ν* are the modulus and Poisson’s ratio of the tested material and $E_{r}$ is the reduced elastic modulus and written as:

(5) $$E_{r}=\frac{\sqrt{\pi }}{2\beta }\frac{S}{\sqrt{A_{c}}}$$

where β=1.034 is the shape constant of the Berkovich tip. Consequently, the elastic modulus and hardness of indented material can be acquired.

In nanoindentation, the indentation strain rate is defined as ${\dot{\varepsilon }}_{i}=\frac{dh}{hdt}$ and can be controlled by setting a constant $\frac{\dot{P}}{P}$. Then the indentation effective shear strain rate can be calculated by the following equation [1,32]:

(6) $$\dot{\gamma }=\sqrt{3}C{\dot{\varepsilon }}_{i}$$

where the parameter *C* = 0.09 [35,36,37].

### 2.3. Combined Shear-Compression Tests

Uniaxial compression, shear and combined shear-compression tests were conducted on PMMA specimens for obtaining its failure loci under different stress states through a universal testing machine (CMT5150A, SUNS, Shenzhen, China). The uniaxial compression and shear tests are traditional test methods and the normal/shear stresses can be easily obtained. However, directly obtaining the stress components of combined loading is difficult through one-dimensional loading. Based on the analysis of the stress state and static equilibrium conditions in the tilted specimen, the normal and shear stress can be respectively expressed as [2,19]:

(7-1) $$\tau_{s}=\tan\theta \cdot \sigma_{n}$$

(7-2) $$\sigma_{n}=\frac{F_{0}}{A}$$

where $\tau_{s}$ is the shear stress in the tilted specimen, and $\sigma_{n}$ is the compressive normal stress on the tilted specimen/compression plates interface. With regard to the shear–compression specimens, the stress distribution on the specimen/compression plate interface is no longer uniform and the normal stress is the average stress calculated by dividing the applied load by the original specimen area [19]. The stress components of PMMA can then be obtained at the yielding point under different stress states, and the yield loci of PMMA can be plotted in stress space for the investigation of its yield criterion.

## 3. Theory

### 3.1. Yielding of PMMA

A yield criterion is a useful tool to describe the mechanical behaviour of materials especially during the plastic segment. Many studies have reported that the yielding of polymers is always affected by different factors, such as hydrostatic pressure, Lode angle and the sign of normal stress [2,10,14,18,38,39,40]. Therefore, the yield behaviour of PMMA under various stress states should be clarified to decompose the contribution of each factor to polymer yielding. The yield criterion can then be constructed based on the physical aspects by considering the quantified parameter effects. Moreover, the physical significance of the parameter may be revealed by degenerating the criterion into a sample stress state such as uniaxial state. Similarly, a parameter can also be determined by substituting the yield stresses of material under a certain stress state into the yield criterion. The von–Mises criterion can be written as:

(8) $$\sqrt{3J_{2}}=\tau_{0}$$

where $J_{2}$ and $\tau_{0}$ represent the second invariant of deviatoric stress tensor $s_{ij}$ and yield strength of materials under pure shear, respectively. $J_{2}$ is defined as:

(9) $$J_{2}=\frac{tr(s_{ij}^{2})}{2}$$

Based on the description of von-Mises criterion, the relation of $\sigma_{n}=\sqrt{3}\tau_{s}$ can be acquired between the normal and shear stress. Without considering the relation between the normal and shear stress, an elliptic criterion [2,3]:

(10) $$\frac{\sigma_{n}^{2}}{\sigma_{0}^{2}}+\frac{\tau_{s}^{2}}{\tau_{0}^{2}}=1$$

was proposed to describe the yield behaviour of polymers. The predicted yield strength was found to agree well with the experimental results. $\sigma_{0}$ is the experimental compression strength. Given the effects of hydrostatic pressure on the yielding of polymers, the first invariant of stress tensor, that is, $I_{1}$, should be considered whilst building the yield criterion of polymers. $I_{1}$ is defined as:

(11) $$I_{1}=tr(\sigma_{ij})$$

where $\sigma_{ij}$ is the stress tensor. As indicated by previous studies [9,41], the PMMA exhibits evident strength differential due to the different yield mechanisms. which indicates that the yielding of PMMA is sensitive to the sign of the normal stress. In other words, the plastic deformation of PMMA is sensitive to the sign of the stress, and the macroscopic yield criterion must be represented by an odd function of the principal values of the stress deviator $s_{ij}$ [42]:

(12) $$J_{2}^{3/2}-cJ_{3}=\tau_{0}^{3}$$

where $J_{3}$ is the third invariant of the deviatoric stress and defined as:

(13) $$J_{3}=\frac{tr(s_{ij}^{3})}{3}$$

The parameter c is a material constant which characterises the SD effects of a material. By introducing the effects of hydrostatic pressure into the criterion, the following equation can be obtained as:

(14) $$f={\left(J_{2}^{3/2}-cJ_{3}\right)}^{1/3}=\tau_{0}+bP$$

where *b* is the pressure sensitivity index, which was found to be equal to 0.23 for PMMA [10] and *P* is hydrostatic pressure and defined as:

(15) $$P=-\frac{1}{3}I_{1}=-\frac{1}{3}(\sigma_{1}+\sigma_{2}+\sigma_{3})$$

where $\sigma_{1}$, $\sigma_{2}$ and $\sigma_{3}$ are the maximum, intermediate and minimum principal stress, respectively. The aforementioned criterion can then be described based on principal stresses as:

(16) $$\begin{array}{l} f={\left(\begin{array}{l} {\left(\frac{1}{3}(\sigma_{1}^{2}+\sigma_{2}^{2}+\sigma_{3}^{2}-\sigma_{1}\sigma_{2}-\sigma_{1}\sigma_{3}-\sigma_{2}\sigma_{3})\right)}^{3/2}\\ -\frac{c}{27}(2\sigma_{1}-\sigma_{2}-\sigma_{3})(2\sigma_{2}-\sigma_{1}-\sigma_{3})(2\sigma_{3}-\sigma_{1}-\sigma_{2}) \end{array}\right)}^{1/3}=\tau_{0}-\frac{b}{3}(\sigma_{1}+\sigma_{2}+\sigma_{3}) \end{array}$$

For the two-dimension stress state, the intermediate principal stress can be ignored and the criterion can be simplified to:

(17) $$f={\left(\begin{array}{l} {\left(\frac{1}{3}(\sigma_{1}^{2}-\sigma_{1}\sigma_{3}+\sigma_{3}^{2})\right)}^{3/2}\\ -\frac{c}{27}(2\sigma_{1}^{3}+2\sigma_{3}^{3}-3(\sigma_{1}+\sigma_{3})\sigma_{1}\sigma_{3}) \end{array}\right)}^{1/3}=\tau_{0}-\frac{b}{3}(\sigma_{1}+\sigma_{3})$$

Regarding the combined shear-compression stress state in the present study, the principal stresses can be calculated by using the following equation:

(18) $$\begin{array}{l} \sigma_{1}\\ \sigma_{3} \end{array}\}=\frac{\sigma_{n}}{2}\pm \sqrt{{\left(\frac{\sigma_{n}}{2}\right)}^{2}+\tau_{s}^{2}}$$

Then, the criterion can be presented by stress components:

(19) $$f={\left({\left(\frac{1}{3}(\sigma_{n}^{2}+3\tau_{s}^{2})\right)}^{3/2}-\frac{c}{27}(2\sigma_{n}^{3}+9\sigma_{n}\tau_{s}^{2})\right)}^{1/3}+\frac{b}{3}\sigma_{n}=\tau_{0}$$

For compression, $\sigma_{n}=-\sigma_{0},\tau_{s}=0$ and Equation (18) can be rewritten as:

(20) $$f={\left({\left(\frac{1}{3}\sigma_{0}^{2}\right)}^{3/2}-\frac{2c}{27}\sigma_{0}^{3}\right)}^{1/3}-\frac{b}{3}\sigma_{0}=\tau_{0}$$

The relationship between $\sigma_{0}$ and $\tau_{0}$ can then be derived as follows:

(21) $$\sigma_{0}=\frac{1}{\left[{\left(\frac{\sqrt{3}}{9}+\frac{2c}{27}\right)}^{1/3}-\frac{b}{3}\right]}\tau_{0}$$

For materials such as glassy polymers, the yield strength $\sigma_{0}$ can be converted to the hardness obtained from the nanoindentation tests by using Tabor’s factor of 3 [30,31,43]:

(22) $$H=3\sigma_{0}=\frac{3}{\left[{\left(\frac{\sqrt{3}}{9}+\frac{2c}{27}\right)}^{1/3}-\frac{b}{3}\right]}\tau_{0}$$

The size effects on the hardness can be analysed based on preceding equation.

### 3.2. Size-Dependent Hardness

Shear transformation-mediated plasticity is a modern understanding regarding the flow of polymers and is based on the deformation mechanism of formation of discrete shear transition areas that locally encompass glide, slip, or shear rotation of chains [32]. Shear transformation zone (STz) has been widely employed to study the deformation of amorphous materials [44]. By applying Arrhenius function [45,46], the relationship between the shear strain rate $\dot{\gamma }$ and shear yield stress $\tau_{s}$ [32] is:

(23) $$\dot{\gamma }=\emptyset_{ss}\gamma^{T}\upsilon_{G}\exp\left(-\frac{\Delta F_{0}}{k_{B}T}\right)\sinh\left(\frac{\gamma^{T}\Omega \tau_{s}}{2k_{B}T}\right)$$

where $k_{B}$ and *T* are Boltzmann constant and absolute temperature, respectively. $\Delta F_{0}$, Ω and $\gamma^{T}$ are activation energy, volume and shear strain of STz, respectively; $\gamma^{T}$= 0.04. $\emptyset_{ss}$ is a parameter and set to be 0.5 for plastic state, and $\upsilon_{G}=10^{10}\;s^{-1}$ is attempt frequency. The activation energy is expressed as:

(24) $$\Delta F_{0}=f(\upsilon ,\beta )\mu {\left(\gamma^{T}\right)}^{2}\Omega$$

where f(υ,β) has a value close to 0.5 for polymer, and μ is shear modulus and expressed as $\mu =\frac{E}{2(1+\upsilon )}$, in which υ is Poisson’s ratio and is equal to 0.38 for PMMA. Then the shear stress $\tau_{s}$ can be derived as:

(25) $$\tau_{s}=\frac{2k_{B}T}{\gamma^{T}\Omega }\left[\ln\left(\frac{2\dot{\gamma }}{{\dot{\gamma }}_{0}}\right)+\frac{f(\upsilon ,\beta )\mu {\left(\gamma^{T}\right)}^{2}\Omega }{k_{B}T}\right]$$

Voyiadjis et al. [32] divided the total shear stress $\tau_{tot}$ into two portions, namely, the shear stress associated with formation of a single STz $\tau_{st}$ and the shear stress associated with the plastic deformation of the highly stressed region $\tau_{local}$:

(26) $$\tau_{tot}=\chi \tau_{st}+(1-\chi )\tau_{local}$$

where χ is the total probability of finding a fertile zone that can undergo discrete shear transformation within the deformation zone. The $\tau_{tot}$ can then be obtained by confirming the volume of STz $\Omega_{st}$ and volume of the plastic deformation of the highly stressed region $\Omega_{local}$. For a 3 sided pyramidal Berkovich tip of 65.3° half angle, $\Omega_{local}$ is equal to 164*h*^3^ (*h* is indentation depth). Therefore, the total shear stress can be expressed as:

(27) $$\tau_{tot}=\frac{2k_{B}T}{\gamma^{T}}\left[\begin{array}{l} \frac{\chi }{\Omega_{st}}\left(\ln\left(\frac{2\dot{\gamma }}{{\dot{\gamma }}_{0}}\right)+\frac{f(\upsilon ,\beta )\mu {\left(\gamma^{T}\right)}^{2}\Omega_{st}}{k_{B}T}\right)\\ +\frac{\left(1-\chi \right)}{164h^{3}}\left(\ln\left(\frac{2\dot{\gamma }}{{\dot{\gamma }}_{0}}\right)+\frac{f(\upsilon ,\beta )\mu {\left(\gamma^{T}\right)}^{2}164h^{3}}{k_{B}T}\right) \end{array}\right]$$

The detailed derivation process of the preceding equation can be found in Section 3.2 of Ref. [32]. Considering Equation (21), hardness can be expressed by indentation depth *h*:

(28) $$H=\frac{3}{\left[{\left(\frac{\sqrt{3}}{9}+\frac{2c}{27}\right)}^{1/3}-\frac{b}{3}\right]}\frac{2k_{B}T}{\gamma^{T}}\left[\begin{array}{l} \frac{\chi }{\Omega_{st}}\left(\ln\left(\frac{2\dot{\gamma }}{{\dot{\gamma }}_{0}}\right)+\frac{f(\upsilon ,\beta )\mu {\left(\gamma^{T}\right)}^{2}\Omega_{st}}{k_{B}T}\right)\\ +\frac{\left(1-\chi \right)}{164h^{3}}\left(\ln\left(\frac{2\dot{\gamma }}{{\dot{\gamma }}_{0}}\right)+\frac{f(\upsilon ,\beta )\mu {\left(\gamma^{T}\right)}^{2}164h^{3}}{k_{B}T}\right) \end{array}\right]$$

Then χ can be derived as:

(29) $$\chi =\frac{\frac{H\cdot \gamma^{T}}{\frac{3}{\left[{\left(\frac{\sqrt{3}}{9}+\frac{2c}{27}\right)}^{1/3}-\frac{b}{3}\right]}\cdot 2k_{B}T}-\frac{1}{164h^{3}}\left(\ln\left(\frac{2\dot{\gamma }}{{\dot{\gamma }}_{0}}\right)+\frac{f(\upsilon ,\beta )\mu {\left(\gamma^{T}\right)}^{2}164h^{3}}{k_{B}T}\right)}{\frac{1}{\Omega_{st}}\left(\ln\left(\frac{2\dot{\gamma }}{{\dot{\gamma }}_{0}}\right)+\frac{f(\upsilon ,\beta )\mu {\left(\gamma^{T}\right)}^{2}\Omega_{st}}{k_{B}T}\right)-\frac{1}{164h^{3}}\left(\ln\left(\frac{2\dot{\gamma }}{{\dot{\gamma }}_{0}}\right)+\frac{f(\upsilon ,\beta )\mu {\left(\gamma^{T}\right)}^{2}164h^{3}}{k_{B}T}\right)}$$

## 4. Results and Discussion

### 4.1. Yield Loci of PMMA

Figure 1 shows the force–displacement curves of PMMA under compression, combined shear-compression and shear. The yield force is found to decrease with increasing tilting angle due to the introduction of shear force. The force component in shear direction can be controlled by the tilting angle. In addition, the shear force component increases with tilting angle. This finding indicates that the stress states of PMMA varies with the tilting angle of specimens. In another word, the mechanical behaviours of PMMA under different combined shear-compression are obtained, and the yield loci of PMMA under different stress states can also be captured as shown in Figure 1. The definition of ‘yielding’ should be explicated firstly, and then the yield strength of PMMA can be obtained. The nominal maximum stress for polymers before the load drop is defined as the yield stress. Therefore, the yield strengths of PMMA studied in the present paper under various stress states as well as the invariants of stress tensor or deviator calculated through the equations in Section 3.1 can be obtained and listed in Table 1. 

**Figure 2:**
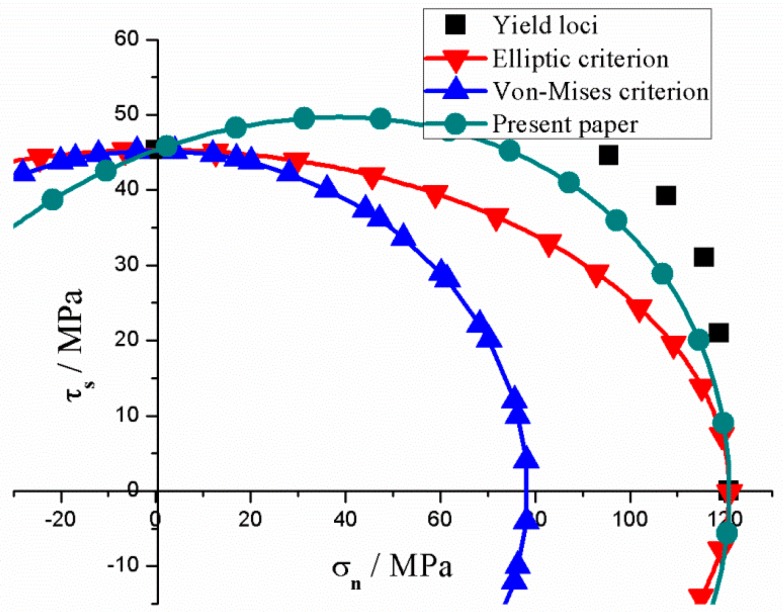
Comparison of experimental yield loci and theoretical models of PMMA in shear–normal stress space.

**Figure 3:**
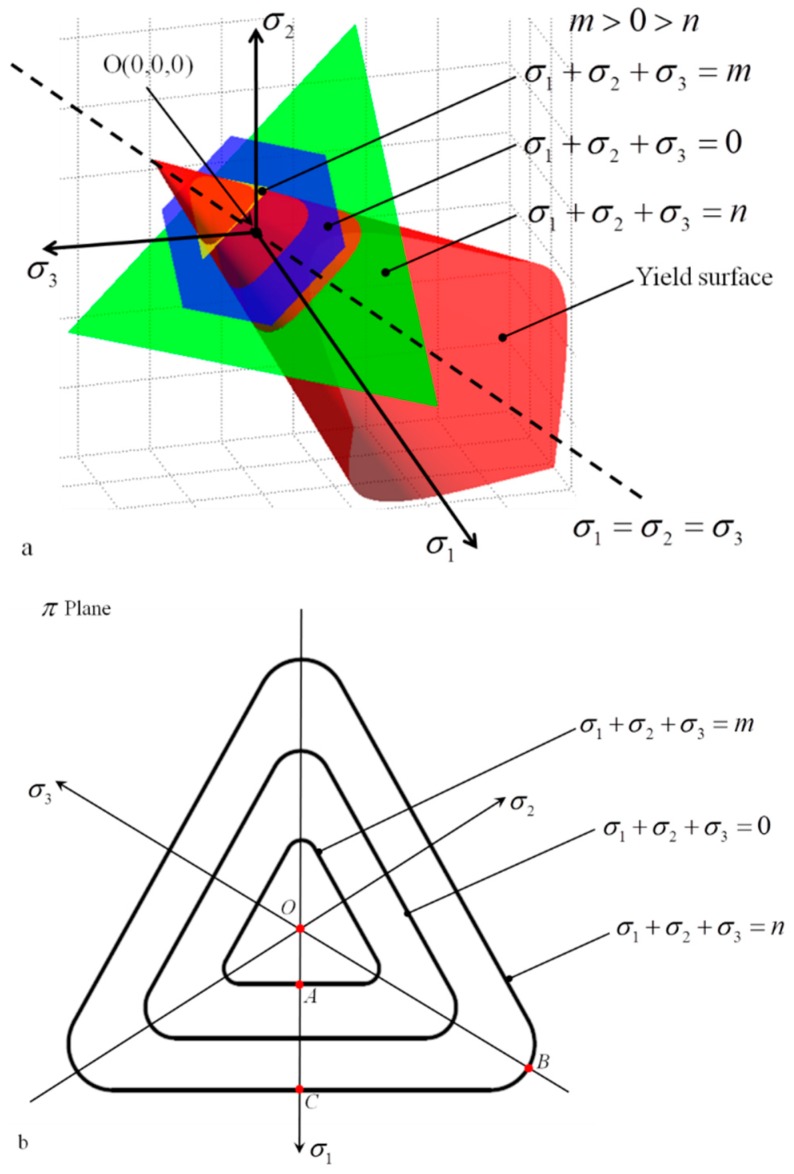
Yield surface in 3–dimension principal stress space (**a**) and π plane (**b**).

Equation (13) has two parameters, and parameter *b* = 0.23 has been experimentally clarified by Prasad [10]. Hence, only parameter *c* remains unknown. Therefore, the stress components ($I_{1}$,$J_{2}$,$J_{3}$) of compression strength were selected to solve the parameter *c* in Equation (13). Moreover, *c* was found to be −1.36 within the numerical range of $\left[-3\frac{\sqrt{3}}{2},3\frac{\sqrt{3}}{4}\right]$ to ensure that the yield criterion satisfies convex analysis [42]. Then the yield loci of PMMA can be plotted in the shear−normal stress space as shown in Figure 2 The yield criterion which considers the pressure sensitivity and SD effects evidently provides the best description of the mechanical behaviour of PMMA. The von−Mises and elliptic criterion underestimate the yield strength of PMMA under combined shear−compression. The yield criterion was plotted in 3−dimension principal stress space and π plane as shown in Figure 3 to intuitively understand this criterion. The surface is closed in the positive direction of $\sigma_{1}=\sigma_{2}=\sigma_{3}$, whereas the opposite is true in the negative direction. This finding implies that pure hydrostatic compression cannot lead to the yielding of PMMA but affects the yield strength linearly. Moreover, the yield strength exhibits increases with decreasing $I_{1}$. Cutting off the yield surface using three planes with different $I_{1}$ (m > 0 > n) values leads to three closed curves as displayed in Figure 3b The size of the closed curve evidently increases with decreasing $I_{1}$ as previously mentioned, thereby indicating the pressure sensitivity. The criterion represents a ‘triangle’ with rounded corners in deviatoric plane which reflects the SD effects. For uniaxial tension strength |AO|, a positive $I_{1}$ can be obtained, and the corresponding yield point *A* is located at the plane of $\sigma_{1}+\sigma_{2}+\sigma_{3}=m$. However, for uniaxial compression |BO|, a negative $I_{1}$ can be calculated which indicates that the corresponding yield point *B* is located at the plane of $\sigma_{1}+\sigma_{2}+\sigma_{3}=n$. Owing to the existence of pressure sensitivity of the yielding, the difference between |AO| and |BO| is the result of the compound action of hydrostatic pressure and SD effects. Therefore, the exact SD effects should be described as |BO|−|AO|−|AC|, and the |AC| represents the pressure sensitivity which can be expressed as: $\frac{b}{3}\left(\left|BO\right|+\left|AO\right|\right)$. Thus, the effects of $I_{1}$ and $J_{3}$ on the yielding of materials, which prove the rationality of the model (Equation (13)) established in Section 3.1 of the present study, must be independently analyzed.

### 4.2. Nanoindentation Measurements of PMMA

**Figure 4:**
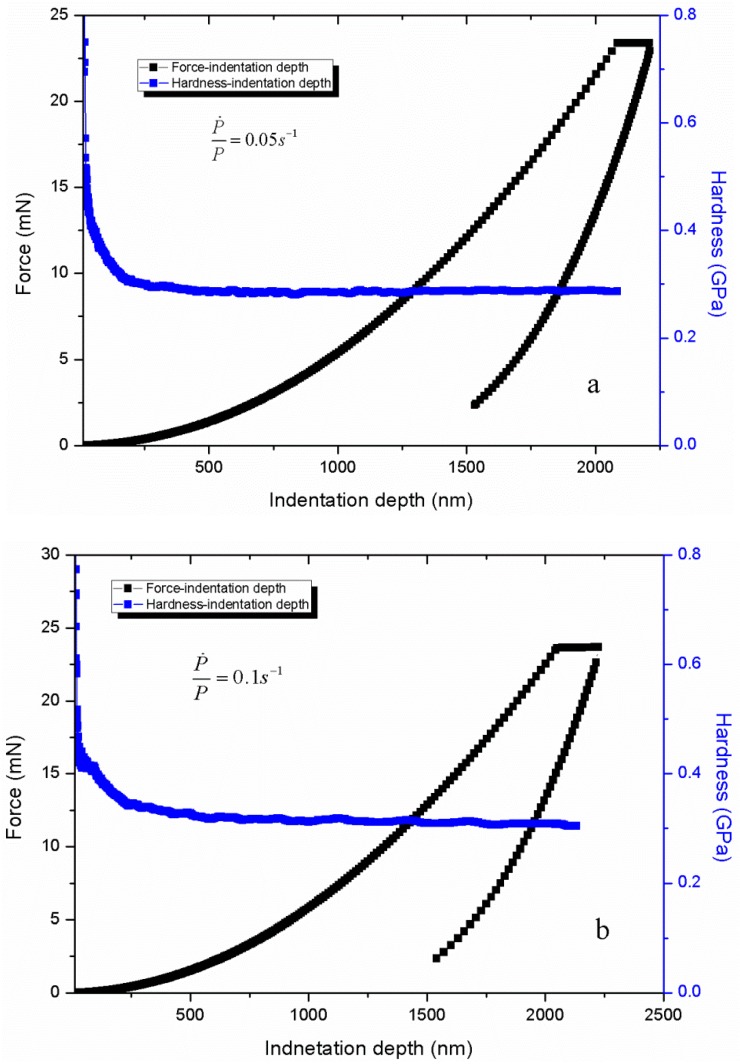

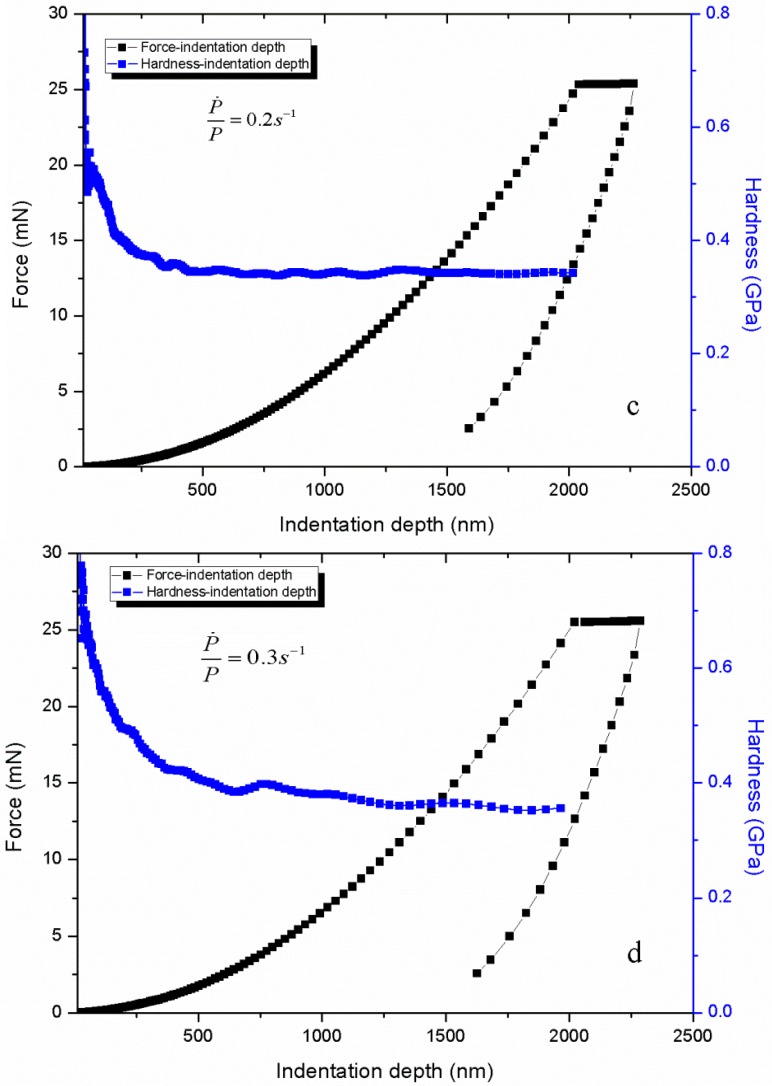
Typical nanoindentation force–depth curves and relation of hardness and indentation depth under different $\frac{\dot{P}}{P}$.

**Figure 5:**
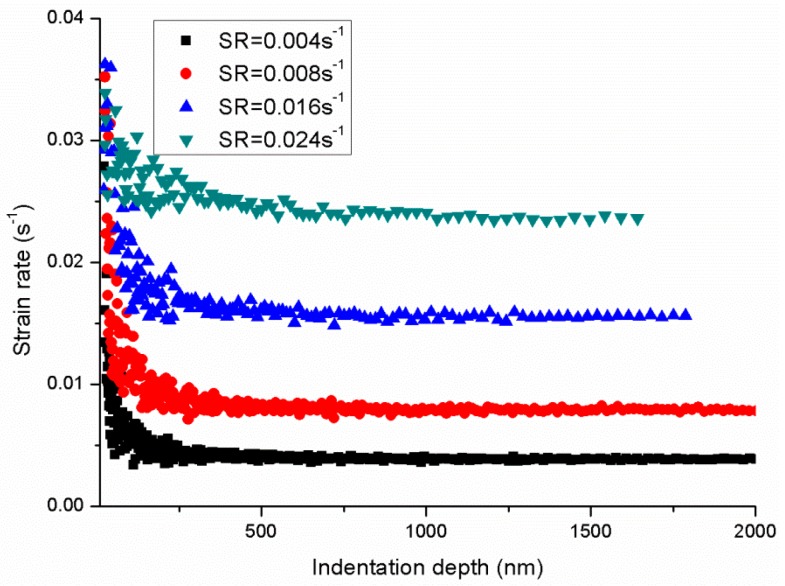
Variation of the indentation strain rate vs depth of PMMA at different indentation strain rates.

**Figure 6:**
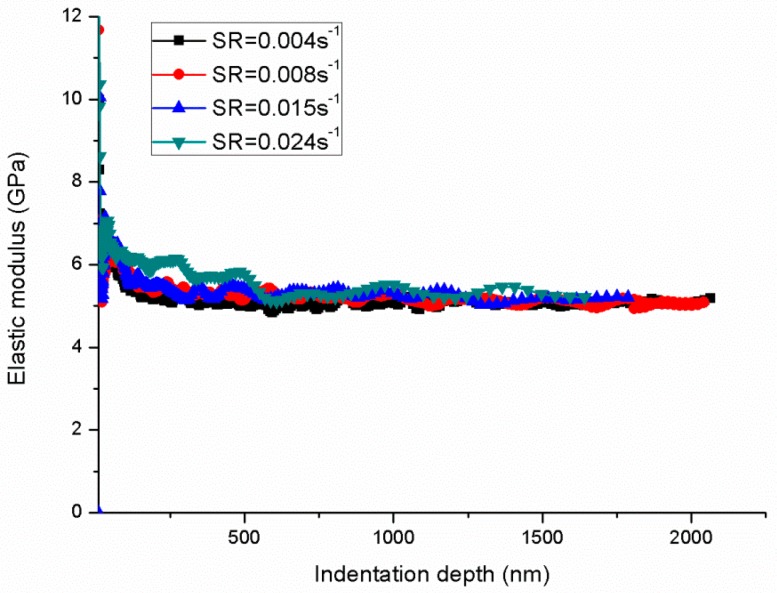
Elastic modulus–depth curves under different indentation strain rates.

Figure 4 shows the nanoindentation force–depth curves and the relation of hardness and indentation depth under different $\frac{\dot{P}}{P}$. The force–depth curve is typical and contains the loading, hold and unloading processes. Then, the indentation strain rate ${\dot{\varepsilon }}_{i}=\frac{dh}{hdt}$ can be calculated through the recorded indentation depth–time data as plotted in Figure 5 The indentation strain rate is evidently not a constant value and decreases as the indentation depth increases. However, all indentation strain rates converge to a constant value, that is, 0.004, 0.008, 0.016 and 0.024 s^−1^ corresponding to different $\frac{\dot{P}}{P}$ sets of 0.05, 0.1, 0.2 and 0.3 s^−1^, respectively. Based on the CSM, the continuous hardness and modulus can be obtained as shown in Figure 4 and Figure 6, respectively. The hardness and modulus exhibit indentation size effects and decrease with increasing depth. The hardness shows remarkable size dependence with values at small depth up to 1.3–2 times of those for deep indentation whereas the ratio for modulus is less than about 1.2. Therefore, the reliability issue in term of hardness and modulus arises from the size effects of indentation measurement. Nanoindentation is known to be a nondestructive method to test the mechanical properties (hardness and modulus) of tested materials, and the properties are obtained by averaging the values of the set indentation depth range based on CSM. Hence, an appropriate indentation depth range should be set for calculating the hardness and modulus. 

### 4.3. Comparison Between the Yield Models

Based on the shear transformation-mediated plasticity, the size of STz $\Omega_{st}$ should be obtained firstly. Then, the χ can be calculated at each indentation depth under a certain strain rate and then the modelled hardness can be obtained through Equation (28). Taken together, the indentation size effects of materials can be investigated through the following steps:

1. Correlate the hardness and shear stress, considering the SD effects and hydrostatic pressure, a yield criterion $f(I_{1},J_{2},J_{3})$ was proposed to describe the yield behaviour of PMMA based on the von-Mises criterion $f(J_{2})$.

2. Calculate the size of STz, $\Omega_{st}$, The $\Omega_{st}$ can be calculated directly using Equation (26) by setting χ equal to 1 represents the total shear stress comprising $\tau_{st}$ results from the formation of STz. By applying different yield criteria, different values of $\Omega_{st}$ would be calculated. Therefore, an appropriate yield model should be selected. 

3. Obtain the relation between χ and indentation depth, By substituting the calculated $\Omega_{st}$ into Equation (26), the χ at each indentation depth during the entire indentation process can be calculated based on an appropriate yield model.

4. Modelled hardness can be calculated with increasing indentation depth, Based on the preceding steps, the necessary parameters, that is, χ and $\Omega_{st}$, can be confirmed and then the hardness can be modelled.

**Figure 7:**
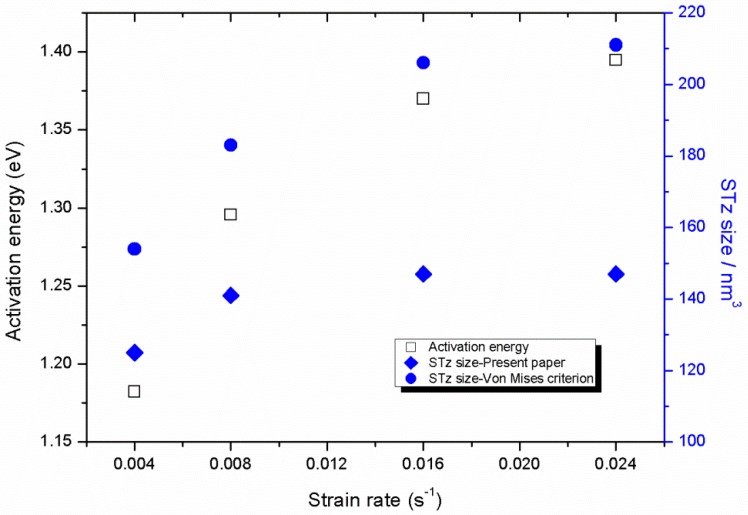
Shear transformation zone (STz) size calculated through different yield models and activation energy under four strain rate.

The von-Mises criterion was selected to calculate the $\Omega_{st}$ to study the difference results from different yield criteria and then compared them with those applied in the proposed model in the present study. The calculated $\Omega_{st}$ are shown in Figure 7 and listed in Table 2, $\Omega_{st}$ calculated through $f(J_{2})$ are evidently below that calculated from $f(I_{1},J_{2},J_{3})$. The $\Omega_{st}$ calculated through $f(I_{1},J_{2},J_{3})$ are all around 120 nm^3^, whereas $\Omega_{st}$ calculated through $f(J_{2})$ are significantly higher than 120 nm^3^. As mentioned by Voyiadjis et al. [1] if PMMA monomers were assumed as the cylinders with radius of 2.85 Å and length of 1.55 Å [47], then the single STz with the $\Omega_{st}$ of 120 nm^3^ is found to contain approximately 3000 monomers, which agrees well with the simulations [48]. Both $\Omega_{st}$ calculated from two criteria exhibit increass with strain rate which indicates the rate sensitivity of $\Omega_{st}$. The activation energy $\Delta F_{0}$ of STz can then be obtained based on Equation (23), and $1k_{B}T$ corresponds to the activation energy of 0.0258 eV. The activation energy of STz can be obtained as displayed in Figure 7 and listed in Table 2. The activation energy evidently increases with strain rate, which is similar to $\Omega_{st}$. Refer to the formula of $\Delta F_{0}$, the activation energy is related to elastic modulus and $\Omega_{st}$. Moreover, $\Omega_{st}$ has been found to have a positive correlation with the strain rate and the elastic modulus as shown in Figure 6 f(υ,β) and $\gamma^{T}$ are constant, hence, $\Delta F_{0}$ also has a positive correlation with the strain rate. 

**Figure 8:**
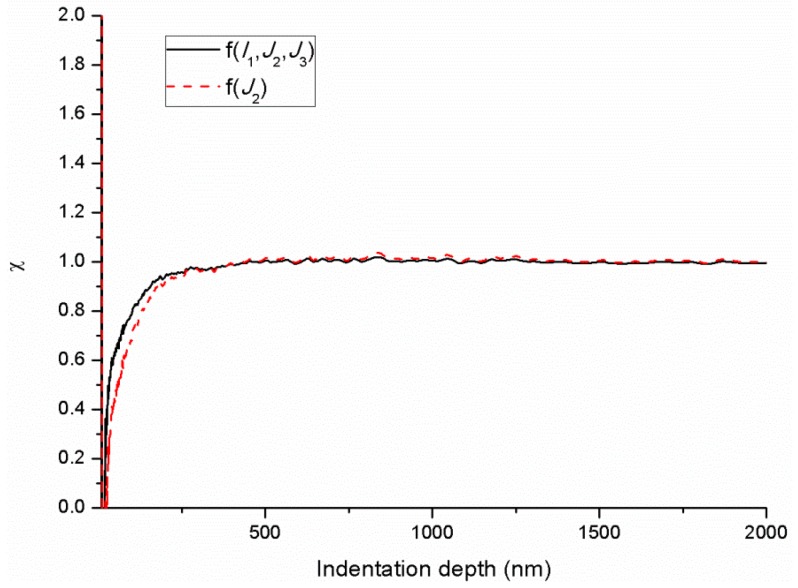
Comparison of the total probability χ of finding a fertile zone that can undergo shear transformation in the deformation zone with depth for different yield criteria of $f(J_{2})$ and $f(I_{1},J_{2},J_{3})$.

**Figure 9:**
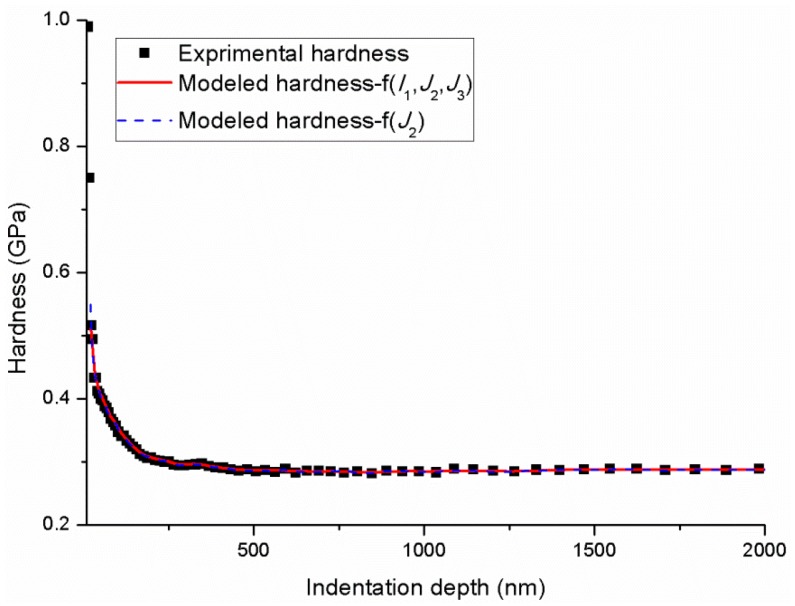
Comparison of the modelled hardness with depth for different yield criteria of $f(J_{2})$ and $f(I_{1},J_{2},J_{3})$.

Figure 8 shows the comparison of the total probability χ of finding a fertile zone that can undergo shear transformation in the deformation zone with depth at the strain rate of 0.004 s^−1^ for different yield criteria of $f(J_{2})$ and $f(I_{1},J_{2},J_{3})$. Both χ for $f(J_{2})$ and $f(I_{1},J_{2},J_{3})$ exhibit an increase at first and then converge to 1. The major difference between the two curves is the rate of χ increases before convergence (the indentation depth of approximately 500 nm), which can be attributed to the different calculated $\Omega_{st}$. At shallow depths, the $\tau_{total}$ mainly comes from $\tau_{local}$ with a small value of χ. As the indentation depth increases, the contributions to the $\tau_{total}$ of STz ($\tau_{st}$) increase, which results in the increasing χ until the indentation depth reaches 500 nm. At this stage, the $\tau_{total}$ mainly comes from $\tau_{st}$ and χ converges to 1. Figure 9 displays the comparison of the modelled hardness with depth for different yield criteria of $f(J_{2})$ and $f(I_{1},J_{2},J_{3})$. Both modelled hardness show excellent agreement with the experimental results. Thus, the forecasting performance of the hardness is inadequate for the evaluation of the reasonability of the selected yield criterion. Therefore, a yield criterion that fully considers the factors influence the yielding of materials should be applied. 

### 4.4. Influence of Strain Rate on PMMA Indentation Size Effects

**Figure 10:**
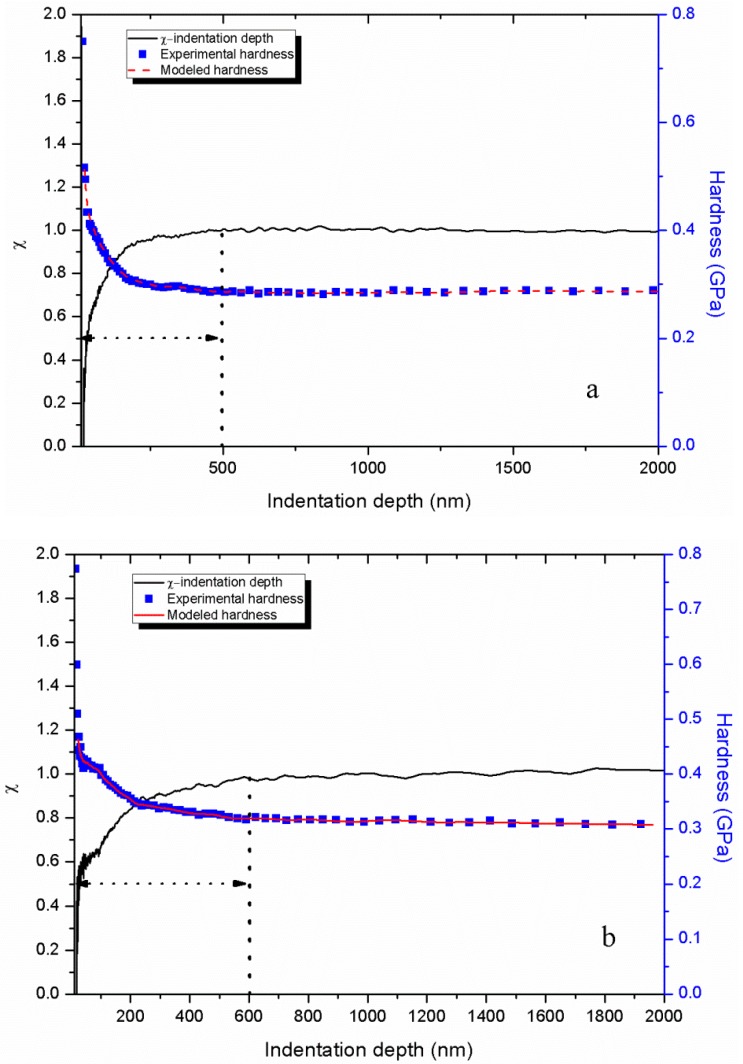

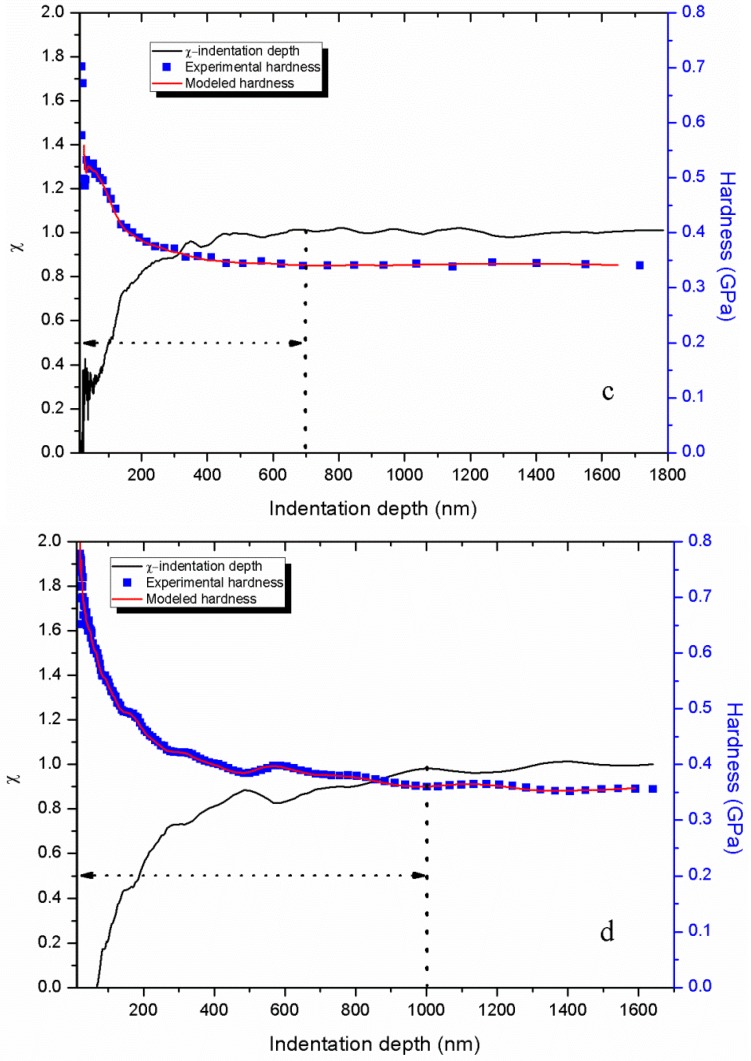
Relation of χ and depth and comparison of modelled hardness and experimental hardness with depth for the yield criteria of $f(I_{1},J_{2},J_{3})$ at different strain rates of 0.004 s^−1^ (**a**), 0.008 s^−1^ (**b**), 0.016 s^−1^ (**c**) and 0.024 s^−1^ (**d**).

The indentation measurements exhibit evident indentation size dependence. From Equation (25), the size effects on the indentation measurements can be attributed to the different ratios of $\tau_{st}$ and $\tau_{local}$ to $\tau_{total}$. At shallow depths, $\tau_{local}$ occupies a high proportion in $\tau_{total}$ and shows up as a lower χ. As indentation depth increases, $\tau_{st}$ provides additional contribution value to $\tau_{total}$ which represents a growing proportion χ of $\tau_{total}$ until a certain indentation depth is reached. This specific $d_{sp}$ depth means $\tau_{local}$ almost has no effect on $\tau_{total}$ anymore. In other words, $d_{sp}$ marks the minimum depth that tested hardness and modulus and could represent its bulk properties. As previously discussed, the $\Omega_{st}$ increases with strain rate. For a large $\Omega_{st}$ under high strain rate, additional depth is needed for a material with a large $\Omega_{st}$ approach its bulk value that size effects disappears. This finding can be attributed to the value of χ for a large $\Omega_{st}$ is smaller than that for a smal $\Omega_{st}$ at the same indentation depth [32]. The $d_{sp}$ can be reasonably inferred to increase with strain rate. Based on the experimental hardness–depth results and calculated $\Omega_{st}$ at different strain rates, the relation of χ and depth at each test with different strain rates can be obtained as shown in Figure 10. Moreover, the modelled hardness agrees well with the experimental results at different loading conditions, which indicates that the model proposed by Voyiadjis et al. [32] shows an excellent performance in describing the indentation size-dependent behaviour of GPs. 

**Figure 11:**
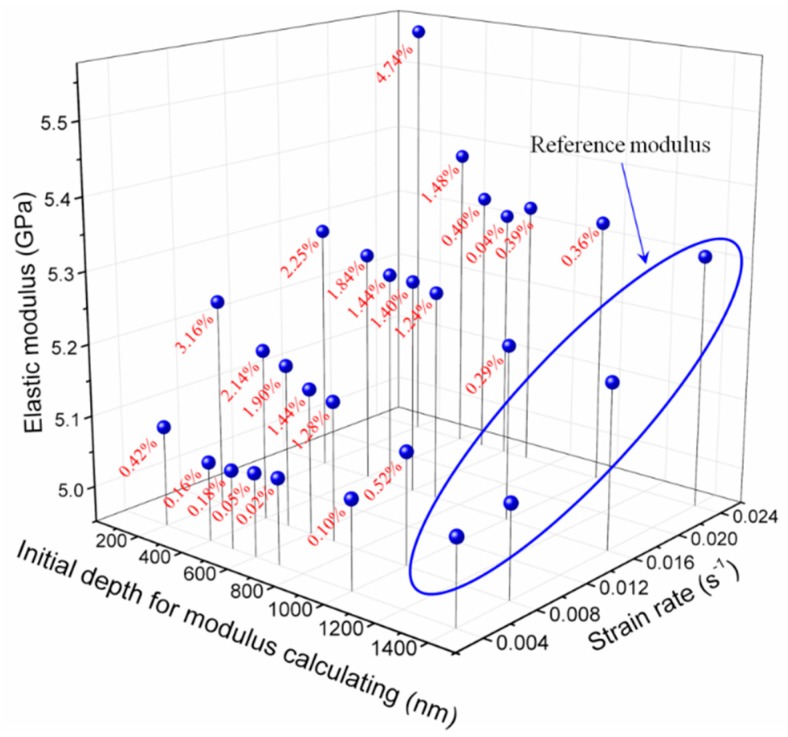
Three-dimensional plots of the relationship of elastic modulus against strain rate and depth range.

**Figure 12:**
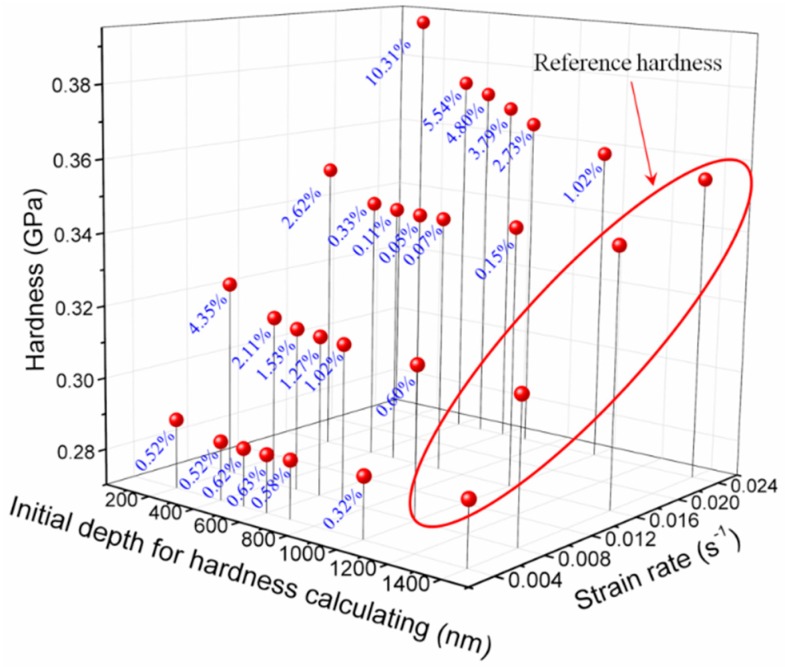
Three-dimensional plots of the relationship of hardness against strain rate and depth range.

Figure 10 evidently shows that $d_{sp}$ increases with strain rate as previously analysed. On the other hand, this indicates that the size effects in amorphous materials can be attributed to the activity of discrete shear transformation units in the deformation zone under indenter. And the discrete shear transformation units are sensitive to strain rate (loading conditions). This means that different depth ranges should be determined under different strain rates for obtaining the reasonable indentation measurements without the size effect. As previously mentioned (Section 4.2), an appropriate indentation depth range should be set for calculating the hardness and modulus based on CSM. Therefore, the average modulus and hardness values (Table 3) from different depth ranges (200–2000 nm, 400–2000 nm, 500–2000 nm, 600–2000 nm, 700–2000 nm, 1000–2000 nm and 1400–2000 nm) under four strain rates were calculated to analyse the effects of depth range. Figure 11 shows the three-dimensional relationship of elastic modulus against strain rate and depth range. The negative correlation between modulus and initial depth for modulus calculation can be observed. This directly reflects the importance of selecting an appropriate indentation depth range for elastic modulus calculation. A parameter η is introduced to describe the difference between the calculated modulus to display the influence of depth range on the modulus intuitive, and η is expressed as:

(30) $$\eta =\left|\frac{E_{D,S}-E_{1400,S}}{E_{1400,S}}\right|\times 100$$

where $E_{D,S}$ is the average value of modulus obtained through the depth range of *D*–2000 nm and strain rate of *S*. Additionally, the average value of modulus $E_{1400,S}$ obtained through depth range of 1400–2000 nm was selected to represent the bulk modulus (Reference modulus) as shown in Figure 11. η clearly decreases with increasing *D*, whereas the opposite tendency is found with strain rate. This phenomenon illustrates the size effects in polymer indentation measurements and the strain rate dependence of indentation size effects. If an error of 1% or 2% is allowed, then the depth range should be set as 1000–2000 nm or 500–2000 nm, respectively, to obtain a responsible elastic modulus. Analogous circumstance can be found in hardness and the three-dimensional illustration of the relationship of hardness against strain rate and depth range is shown in Figure 12. From an overall perspective, the hardness increases with strain rate and decreases with increasing initial depth for hardness calculation. A parameter marked as ψ can be used to characterise the difference between the values of hardness obtained from different depth ranges under four strain rates and expressed as:

(31) $$\psi =\left|\frac{H_{D,S}-H_{1400,S}}{H_{1400,S}}\right|\times 100$$

where $H_{D,S}$ is the average value of hardness obtained through the depth range of *D*–2000 nm and strain rate of *S*. Additionally, the average value of hardness $H_{1400,S}$ obtained through the depth range of 1400–2000 nm was selected to represent the bulk hardness (reference hardness) as shown in Figure 12. The parameter ψ shows evident negative correlation to *D* which indicates the significant size effects in PMMA hardness. The phenomenon of ψ increases with strain rate, which indicates that size effects in hardness is also strain rate sensitive. The depth range can then be set based on ψ under different conditions. For example, the depth range should be set as 1000–2000 nm corresponding to the error of 1%. Taken together, the indentation size effects in PMMA is inevitable and exhibits strain rate dependence. Hence, an appropriate indentation depth range should be selected for obtaining reasonable measurements. Within the range tested in the present study, the initial depth for modulus/hardness calculation should be set as 1000 nm.

## 5. Conclusions

An indentation size effect model is proposed in the present study based on the yield criterion involving SD effects, pressure sensitivity, and shear transformation-mediated plasticity. Through the combined loading experiments, the yield behaviour of PMMA is found to be sensitive to the sign of normal stress and hydrostatic pressure. The SD effects are characterised by applying an odd function of the principal values of the stress deviator. Pressure sensitivity is characterised by introducing *P* and controlled by parameter *b*. By adopting the shear transformation-mediated plasticity to indentation results, the size dependence behaviour of PMMA under different strain rates is modelled. The size effects in amorphous materials can be attributed to the activity of discrete shear transformation units in the deformation zone under indenter. Additionally, the volume of single STz is found to increase with strain rate, which results from the increasing indentation depth, represents the bulk properties (size effects disappear) as strain rate increases. An appropriate indentation depth range (approximately 1000 nm) is suggested for calculating the hardness and modulus based on the analysis of the indentation test results.

## Figures and Tables

**Table 1:** Stress components and invariants of PMMA under different loading conditions at yielding.

Stress Component	Compression (MPa)	Combined Shear-Compression (MPa)				Shear (MPa)
		10°	15°	20°	25°	
$\sigma_{n}$	−120.92	−118.85	−115.72	−107.70	−95.61	–
$\tau_{s}$	–	20.96	30.01	39.20	44.58	45.31
$\sigma_{1}$	–	3.59	7.78	12.76	17.56	45.31
$\sigma_{2}$	–	–	–	–	–	–
$\sigma_{3}$	−120.92	−122.44	−123.50	−120.46	−113.17	45.31
$s_{1}$	40.31	43.20	46.36	48.66	49.43	45.31
$s_{2}$	40.31	39.62	38.57	35.90	31.87	0
$s_{3}$	−80.61	−82.82	−84.93	−84.56	−81.30	−45.31
$I_{1}$	−120.92	−118.85	−115.72	−107.7	−95.61	0
$\sqrt{J_{2}}$	69.81	71.75	73.65	73.50	70.95	45.31
$\sqrt[{3}]{J_{3}}$	−50.78	−52.14	−53.35	−52.86	−50.41	0

**Table 2:** Parameters of indentation size effects under different strain rate.

Strain Rate /s^−1^	STz-Size $f(J_{2})/{\mathrm{nm}}^{3}$	STz-Size $f(I_{1},J_{2},J_{3})/{\mathrm{nm}}^{3}$	Activation Energy /eV
0.004	154	125	1.182
0.008	183	141	1.295
0.016	206	147	1.370
0.024	211	147	1.395

**Table 3:** Average modulus and hardness values calculated from different depth ranges under four strain rates.

Depth Range/Nm	Elastic Modulus/GPa				Hardness/GPa			
	0.004 s^−1^	0.008 s^−1^	0.016 s^−1^	0.024 s^−1^	0.004 s^−1^	0.008 s^−1^	0.016 s^−1^	0.024 s^−1^
200–2000	5.090	5.240	5.298	5.558	0.289	0.324	0.351	0.392
400–2000	5.060	5.188	5.277	5.385	0.286	0.317	0.343	0.375
500–2000	5.059	5.175	5.257	5.327	0.286	0.351	0.343	0.372
600–2000	5.066	5.152	5.254	5.308	0.286	0.314	0.342	0.368
700–2000	5.070	5.145	5.246	5.327	0.286	0.313	0.342	0.365
1000–2000	5.073	5.105	5.197	5.325	0.287	0.312	0.342	0.359
1400–2000	5.068	5.079	5.182	5.306	0.288	0.310	0.342	0.355
